# Response time variability and response inhibition predict affective problems in adolescent girls, not in boys: the TRAILS study

**DOI:** 10.1007/s00787-012-0260-2

**Published:** 2012-02-22

**Authors:** Patricia A. M. van Deurzen, Jan K. Buitelaar, J. Agnes Brunnekreef, Johan Ormel, Ruud B. Minderaa, Catharina A. Hartman, Anja C. Huizink, Anne E. M. Speckens, A. J. Oldehinkel, Dorine I. E. Slaats-Willemse

**Affiliations:** 1 Department of Psychiatry, Donders Institute for Brain, Cognition and Behavior, Radboud University Nijmegen Medical Centre, 966, P.O. Box 9101, 6500 HB Nijmegen, The Netherlands; 2 Karakter Child and Adolescent Psychiatry, University Centre, Nijmegen, The Netherlands; 3 Interdisciplinary Center for Psychiatric Epidemiology, University Medical Center Groningen, University of Groningen, Groningen, The Netherlands; 4 Department of Psychiatry, University Medical Center Groningen, University of Groningen, Groningen, The Netherlands; 5 Behavioural Science Institute, Radboud University Nijmegen, Nijmegen, The Netherlands; 6 Faculty of Behavioral and Social Sciences, University of Amsterdam, Amsterdam, The Netherlands; 7 Department of Cognitive Neuroscience, Donders Institute for Brain, Cognition and Behavior, Radboud University Nijmegen Medical Centre, Nijmegen, The Netherlands

**Keywords:** Depression, Affective problems, Neuropsychology, Gender, Adolescence, Cohort

## Abstract

The present study examines the relationship between neurocognitive functioning and affective problems through adolescence, in a cross-sectional and longitudinal perspective. Baseline response speed, response speed variability, response inhibition, attentional flexibility and working memory were assessed in a cohort of 2,179 adolescents (age 10–12 years) from the TRacking Adolescents’ Individual Lives Survey (TRAILS). Affective problems were measured with the DSM-oriented Affective Problems scale of the Youth Self Report at wave 1 (baseline assessment), wave 2 (after 2.5 years) and wave 3 (after 5 years). Cross-sectionally, baseline response speed, response time variability, response inhibition and working memory were associated with baseline affective problems in girls, but not in boys. Longitudinally, enhanced response time variability predicted affective problems after 2.5 and 5 years in girls, but not in boys. Decreased response inhibition predicted affective problems after 5 years follow-up in girls, and again not in boys. The results are discussed in light of recent insights in gender differences in adolescence and state–trait issues in depression.

## Introduction

Affective problems and neurocognitive problems co-occur in depression but the relationship between these domains is still poorly understood. The characteristic ‘diminished ability to think or concentrate, or indecisiveness’ is a criterium of Major Depressive Disorder (MDD), next to criteria that represent affective and somatic problems [1]. To better understand the phenomenology and etiology of depression, researchers have been investigating the neurocognitive profile of depression. They found that adult patients with MDD show neurocognitive impairments in attentional and executive functioning, short-term memory, working memory and psychomotor speed [2, 3].

Data about the presence of neurocognitive impairments in depressed adolescents are scarce and inconclusive. MDD and Dysthymic Disorder (DD), i.e., a more chronic state of depression during at least 1 year, have been found to be associated with impaired spatial working memory in adolescent girls [4]. Baseline response speed was impaired in a sample of adolescent girls with MDD [4], but not in a mixed sample of boys and girls from the same age group [5]. Both MDD and DD appeared to be associated with impaired verbal working memory [5] and with reduced speed on tasks that appealed to attentional flexibility [6]. Response inhibition deficits were not identified on a Go/No go Task [5, 7], whereas performance on the Stroop Task indicated impaired response inhibition and interference control [7]. Interference control was not shown with the Flanker task [8], and whereas one study identified an enhanced response time variability [7], another study did not [5].

The discrepancies between these study results may be explained by variation in neurocognitive tasks administered, and by differences in sample characteristics, such as variation in the severity of depression of the participants and variation in gender. Despite these discrepancies, a strikingly consistent finding is that neurocognitive impairments were primarily observed in girls but not in boys with depression. Further, it is remarkable that the association between neurocognitive functioning and depression has mainly been studied in cross-sectional case–control designs in which neurocognitive performance of clinically depressed patients was compared to that of non- or never-depressed controls. Case–control studies are designed to indicate associations, i.e., coincidence of depression and neurocognitive impairments, but impede conclusions about causal inferences. Longitudinal studies are needed to study whether neurocognitive impairments lead to depression, or alternatively, whether depression leads to neurocognitive impairments.

A framework that is commonly intertwined with the cause–coincidence distinction is the state–trait discussion. The state–trait dichotomy originates from distinguishing stable personality traits and fluctuating state emotional reactions [9]. Applying the state–trait dichotomy to depression, neurocognitive impairment could represent a trait factor or neurocognitive marker that is already present before depression develops and will be present even after the depressive episode. This trait factor could be an early expression or one of the component causes of depression. Alternatively, neurocognitive impairment could be a state factor, coincidental with depression but not causally related, or could be due to causal factors that are shared between depression and neurocognitive impairment.

Some evidence from adult clinical populations fits the ‘trait’ idea in that some neurocognitive impairments continue to be present when depression is in remission [10–14]. However, impairments in remitted patients may be a consequence of a depressive episode (a ‘scar effect’) or a residual symptom (‘state effect’) rather than a premorbid trait marker [15]. Moreover, full recovery of neurocognitive functioning upon remission of adult MDD has also been reported, e.g., on verbal memory tasks [16–18], measures of attention [16, 19], and verbal fluency [16]. That supports the idea that impaired neurocognitive functioning may be at least a state effect [20] for some of the neurocognitive impairments.

Neurocognitive functioning in remitted MDD patients may yield evidence for state or trait characteristics or for scar effects, but not for causality. A study design that is more likely to generate evidence for causality is a longitudinal prospective cohort study. Cohort studies that have addressed neurocognitive impairment as a potential premorbid marker for depression are sparse and assessed only two neurocognitive functions. Speed of information processing appeared not to be a neurocognitive marker of depression after 2-year follow-up. However, episodic memory predicted depression after 2-year follow-up in a population-based adult female sample [20] and after a 3-year follow-up in a population-based adult sample with both males and females. Nevertheless, episodic memory would not be recommended for neurocognitive screening because of its low specificity and sensitivity [20, 21]. Castaneda and others [2] suggested that more prospective studies starting from young adulthood or even earlier are required to further address this issue.

Impaired neurocognitive functioning may not only be related to MDD, but may already arise with subclinical affective problems. In adolescence, subclinical affective problems are widespread, with prevalence rates ranging from 15 to 40% [22]. These affective problems include changes in sleeping and eating patterns, feeling worthless and having suicidal ideations. Impaired neurocognitive functioning is considered to be mediating the functional adaptation in depression [23, 24]. Functional adaptation is already worsened with as little as one affective problem compared with having no affective problems [25]. Accordingly, impaired neurocognitive functioning may already co-occur with subclinical affective problems. The predictive association between neurocognitive functioning and affective problems in young adolescents may be different from adults for several reasons. Compared to adulthood, adolescence is a time of substantial neurobiological changes that subserve higher cognitive functions, reasoning, interpersonal interactions, cognitive control of emotions, risk-versus-reward appraisal and motivation. These changes may play a role in the susceptibility to the development of affective problems [26–28].

The susceptibility for affective problems seems to differ in girls compared to boys. The female–male prevalence ratio changes from 1:1 prepuberal to 2:1 after puberty [27]. This changing ratio may for at least a part be attributed to hormonal changes that occur during adolescence [26, 27, 29–31]. Furthermore, just like in adults, gender differences in neurocognitive performance have repeatedly been observed in adolescents, in particular a female superiority in processing speed [32, 33] and a male superiority in perceptual analysis and working memory [33, 34]. Impaired neurocognitive functioning in adolescent MDD patients has predominantly been shown in samples of girls [4, 5].

The objective of the present study was to examine the cross-sectional and longitudinal association between neurocognitive functioning and affective problems in a large unselected cohort of adolescents, with three assessment waves. Our first hypothesis was that neurocognitive functioning may be associated with affective problems in adolescent girls, but not in boys. Based on previous research we expected gender differences in neurocognitive functioning and possibly in the association between neurocognitive functioning and affective problems. Our second hypothesis was that neurocognitive functioning in a population-based sample of 10- to 12-year-old adolescents would predict affective problems after 2.5- and 5-year follow-up. Gender was used as a stratifying variable because of expected gender differences in neurocognitive functioning in relation to affective problems. Furthermore, the incidence of affective problems in adolescent girls was expected to gradually outnumber the incidence in boys during the study period.

## Method

### Sample

The TRacking Adolescents’ Individual Lives Survey (TRAILS) is a prospective cohort study of Dutch adolescents, which aims to study the development of mental health from early adolescence into adulthood. Adolescents will be assessed every 2–3 years from age 10–12 until at least the age of 24. The study has been approved by the Dutch Central Committee on Research Involving Human Subjects (CCMO) and informed consent has been appropriately obtained. The present study involved data from the first, second and third assessment wave of TRAILS. The first assessment (*N* = 2,230, mean age = 11.1, range 10–12 years, 50.7% girls) was a baseline assessment, the second assessment (*N* = 2,087, mean age = 13.6, range 12–15 years, 51.2% girls) was 2.5 years after baseline, and the third assessment (*N* = 1,819, mean age = 16.3, range 14.5–18.5 years, 52.3% girls) was 5 years after baseline assessment. Detailed information about the TRAILS sample is provided elsewhere [35, 36]. For the present study, participants were selected when self-reported problem behavior and neurocognitive functioning data were available at the first assessment (*n* = 2,179; 97.7% of the TRAILS sample). There are no indications of differences in the prevalence of affective problems or other problem domains between study participants and a reference group of non-participants from the original school population [35, 36]. However, the eight adolescents with unavailable data on problem behavior (including affective problems) performed worse on the neurocognitive measure response time variability (a standard deviation of 2.8 vs. 1.8 ms, *t* = −3.3; *p* = 0.001) than those with full information available. Attrition at wave two was not associated with baseline affective problems, but showed some associations with neurocognitive functioning in that study dropouts at wave 2 showed lower baseline speed at wave 1 than participants (*p* = 0.031). Attrition at wave 3 was associated with less baseline affective problems and worse performance on all baseline neurocognitive functions (except for attentional flexibility) in dropouts compared to participants (all *p*s > 0.04). Given that baseline affective problems predict affective problems at follow-up, attrition may have caused minor bias in an overestimation of the association between baseline speed at wave 1 and affective problems at wave 2, and an underestimation of the association between all neurocognitive functions (except for attentional flexibility) at wave 1 and affective problems at wave 3.

### Procedure and measures

#### Neurocognitive functioning

Neurocognitive functioning was assessed at baseline wave 1. Based on previous research [4, 5, 7] we included measures that cover a broad range of neurocognitive functioning, i.e., information processing. The following five measures from four tasks of the Amsterdam Neuropsychological Tasks Program (ANT) [37] were selected: (1) baseline response speed (Baseline Speed task); (2) response speed variability (Sustained Attention dot patterns task); (3) response inhibition (Visual Attention Set Shifting task), (4) attentional flexibility (Visual Attention Set Shifting task); and (5) working memory (Memory Search Letters task).

In the Baseline Speed task, a white fixation cross that is presented in the center of the screen changes into a white square after a random time interval. Children were instructed to respond to this stimulus change as fast as possible by pressing a mouse button with the index finger. Baseline response speed is calculated as the mean reaction time (RT), providing a baseline measure of the child’s speed of responding to the occurrence of a stimulus.

In the Sustained Attention dot patterns task, 600 dot patterns with 3, 4 of 5 dots are successively presented during approximately 15 min. Children are required to respond to 4 dots by pressing the mouse button with their dominant hand (‘yes’ response) and to respond to 3 or 5 dots by pressing the mouse button with their non-dominant hand (‘no’ response). Response speed variability is computed as the within-subject standard deviation of the mean RT and may therefore be interpreted as a measure of response stability in a continuous task performance.

In the Visual Attention Set Shifting task, a horizontal bar consisting of ten squares is permanently presented in the center of the screen. In each trial, a colored square moves across the bar in a randomly varied direction (either to the left or to the right). The task consists of three parts, each requiring different responses. Part 1 requires spatially compatible responses: children are instructed to copy the direction of the movement of a green-colored square (movement to left requires pressing left mouse button and movement to right requires pressing right mouse button). Part 2 requires spatially incompatible responses: children are instructed to ‘mirror’ the direction of the movement of a red-colored square (movement to left requires pressing right mouse button and movement to right requires pressing left mouse button). In part 3 the color of the moving square randomly alternates between green and red. When the color of the square is green, a spatially compatible response is required (as in part 1). When the color of the square is red, a spatially incompatible response is required (as in part 2). Response inhibition is computed by subtracting the mean RT of part 1 (stimulus–response compatible situation) from the mean RT of part 2 (stimulus–response incompatible situation). Attentional flexibility reflects the central neurocognitive ability to mentally switch between two competing and unpredictable response sets. It is computed by subtracting the mean RT of the compatible responses of part 1 from the mean RT of the compatible responses of part 3.

The Memory Search Letter task comprises three parts. Before each part, children are instructed to memorize respectively one, two or three target consonants. The subsequently presented display sets consist of four consonants in each trial. Trials that contain the complete target set require a ‘yes’ response (pressing mouse button with dominant hand). Trials that contain none of the target letters or an incomplete target set require a ‘no’ response (pressing mouse button with non-dominant hand). Working memory capacity is computed by subtracting the mean RT in response to target trials of part 1 (requiring memorization and processing of one consonant) from the mean RT in response to target trials of part 3 (requiring memorization and processing of the combination of three consonants). More detailed descriptions of the tasks can be found elsewhere [38–40].

#### Behavior problems

Behavior problems including affective problems were assessed at the first (baseline) assessment wave (age 10–12), the second wave (age 12–15), and the third wave (age 15–18) by the Youth Self Report (YSR/11–18) [41]. A self-report questionnaire was used because it has been shown that parents tend to underreport their children’s depression and children may provide a more valid description of their emotional states than parent reports [42–44]. This questionnaire consisted of 112 items that assessed the severity of problem behaviors on a 3-point scale (range 0–2). Items measuring behavior that relates strictly to the classification system of the Diagnostic and Statistical Manual of Mental Disorders yielded six DSM-scales of which one contained affective problems (13 items) [45]. Content validity of the factor affective problems on the YSR is good [46] and diagnostic accuracy is high [47]. The mean item score on the DSM-scale affective problems was calculated.

### Analyses

We defined scores on reaction time (RT) on the neurocognitive tasks with an absolute z-score greater than or equal to 4 as outliers [48] and removed these from further analyses. Depending on the neurocognitive variable, the amount of outliers ranged from 4 to 11 (0.01–0.04%). Moreover, we excluded participants performing at a chance level of accuracy, i.e., making 50% or more errors on any of the relevant task conditions. Depending on the neurocognitive variable, the amount of excluded participants ranged from 0 to 62 (0.0–2.8%). In the descriptives, affective problems for boys and girls at wave 1, 2, and 3 were tested on correlation and differences. Neurocognitive functions for boys and girls were tested on differences.

To test the hypotheses, regression models for boys and girls separately were built for (1) baseline response speed; (2) response speed variability; (3) response inhibition; (4) attentional flexibility; and (5) working memory. The influences of neurocognitive variables on affective problems were analyzed in separate regression models. This enabled examining the influence of neurocognitive variables unconditional of other neurocognitive variables. In the first set of five regression models, baseline affective problems (T0) were predicted by baseline neurocognitive functioning. In the second set of regression models, affective problems at T1 (2.5 years) were predicted by baseline neurocognitive functioning, and in the third set of regression models, affective problems at T2 (5 years) were predicted by baseline neurocognitive functioning. In the prediction of affective problems at T1 and T2, a secondary correction for baseline affective problems was performed. Finally, the relation between affective problems and error rates on the sustained attention dot patterns task, the visual attention set shifting task, and the memory search letter task was tested by correlation; tests were two-sided. To reduce the chance of type I errors (false-positives) a powerful Holm–Bonferroni correction was applied, taking account of the five different main associations that were studied (*k* = 5; *α* = 0.05/*k* = 0.01). After ordering the *p* values, the smallest *p* value was compared to *α*/*k* (so if *k* = 5, *α* = 0.05/*k* = 0.01), the second *p* value was compared to *α*/*k* − 1, the third to *α*/*k* − 2, etc. [49].

## Results

### Descriptive results

Group characteristics regarding the DSM-scale affective problems for girls and boys separately at baseline are presented in Table 1, including associations and differences between affective problems in boys and girls at the three assessment waves. The mean item score on the DSM-scale affective problems on the YSR was 0.30 (SD = 0.25) for girls and 0.29 (SD = 0.25) for boys (*p* > 0.05) at baseline. Affective problems in girls increased significantly from T0 to T1 to T2 (*p* < 0.01 and *p* < 0.005). Affective problems in boys decreased from T0 to T1 and stabilized from T1 to T2 (*p* < 0.001 and *p* = 0.25). Consequently, at T1 and T2, girls reported more affective problems than boys (*p* < 0.001).

**Table 1:** Descriptives (mean, SD, nonparametric Kendall’s Tau correlations) of affective problems at baseline, follow-up after 2.5-year and follow-up after 5 years, change is severity of affective problems in boys and girls (mean difference score and descriptives (mean, SD, *t* test) of neurocognitive performance of boys and girls

Affective problems [*N*, mean (SD)]			Independent *t* test	*p* value	Kendall’s Tau					
	Girls	Boys			Girls			Boys		
					Baseline	2.5 years	5 years	Baseline	2.5 years	5 years
Baseline (T0)	1,114, 0.30 (0.25)	1,073, 0.29 (0.25)	1.22	0.23	1.00			1.00		
2.5 years (T1)	1,072, 0.32 (0.29)	1,019, 0.22 (0.22)	8.76	<0.001	0.35*	1.00		0.37*	1.00	
5 years (T2)	883, 0.35 (0.30)	777, 0.22 (0.22)	10.42	<0.001	0.25*	0.38*	1.00	0.27*	0.39*	1.00

Change in severity of affective problems in boys and girls										
	Girls	Boys	Dependent *t* test	*p* value						
T1–T0	0.02	−0.06	2.6 and −7.2	0.009 and < 0.001						
T2–T0	0.05	−0.07	4.9 and −7.2	<0.001 and < 0.001						
T2–T1	0.03	−0.01	3.2 and −1.1	0.001 and 0.254						

Neurocognitive performance at baseline (wave 1) [mean (SD)]						
	Girls	Boys			Independent *t* test	*p* value
Baseline response speed	333 (49)	329 (42)			1.89	0.059
Response time variability	1.66 (0.86)	1.86 (0.97)			−5.19	<0.001
Response inhibition	247 (19)	272 (21)			−2.94	<0.005
Attentional flexibility	653 (25)	616 (25)			3.49	<0.001
Working memory	492 (26)	574 (31)			−6.80	<0.001

* *p* < 0.001

Neurocognitive performance in boys and girls differed significantly, see Table 1 for details. Boys outperformed girls in attentional flexibility (*p* < 0.001), whereas girls showed less response speed variability (*p* < 0.001), better response inhibition (*p* < 0.005) and a better working memory function (*p* < 0.001) than boys. There were no differences between boys and girls in baseline response speed (*p* = 0.06). Mean error rates on the tasks ranged from 6 to 19 and did not differ between boys and girls (*p*s > 0.05). Faster reaction times were associated with higher error rates on the Sustained attention dot patterns task (girls, *r* = −0.21; boys, *r* = −0.15; *p*s < 0.001), indicating an accuracy trade-off, but not on the memory search letter task and visual attention set shifting task (*p*s > 0.05).

### Affective problems and neurocognitive functioning

Results of the regression models are shown in Table 2. The cross-sectional analyses indicated that affective problems were associated with a lower baseline response speed (*β* = 0.08, *p* = 0.001), more variability in response time (*β* = 0.10, *p* = 0.002), lower working memory capacity (*β* = 0.08, *p* = 0.008) and lower response inhibition (*β* = 0.08, *p* = 0.012) in girls. In boys, affective problems were not associated with neurocognitive performance (all *p* values > 0.01).

**Table 2:** Summary of results of regression models of neurocognitive performance (*β*) on affective problems at baseline, after 2.5- and 5-year follow-up, taking account of gender

Neurocognitive variable	Girls		Boys	
	Unadjusted	Adjusted for baseline affective problems	Unadjusted	Adjusted for baseline affective problems
Association with affective problems at baseline				
Baseline response speed				
*β*	0.077**	**–**	0.048	**–**
*p*	**0.001**		0.117	
Response time variability				
*β*	0.095**	**–**	0.036	**–**
*p*	**0.002**		0.242	
Response inhibition				
*β*	0.076*	**–**	−0.028	**–**
*p*	**0.012**		0.366	
Attentional flexibility				
*β*	0.035	**–**	−0.026	**–**
*p*	0.255		0.393	
Working memory				
*β*	0.079**	**–**	0.013	**–**
*p*	**0.008**		0.665	
Prediction of affective problems after 2.5-year follow-up				
Baseline response speed				
*β*	0.050	0.001	0.03	0.016
*p*	0.105	0.965	0.305	0.574
Response time variability				
*β*	0.087**	0.029	0.034	0.018
*p*	**0.005**	0.287	0.267	0.552
Response inhibition				
*β*	0.054	0.019	0.004	0.019
*p*	0.082	0.468	0.906	0.492
Attentional flexibility				
*β*	0.013	0.008	−0.018	−0.006
*p*	0.668	0.781	0.563	0.820
Working memory				
*β*	0.049	−0.002	0.028	0.019
*p*	0.113	0.953	0.380	0.501
Prediction of affective problems after 5-year follow-up				
Baseline response speed				
*β*	0.073*	0.046	−0.09	−0.17
*p*	0.030	0.151	0.810	0.608
Response time variability				
*β*	0.082*	0.054	0.058	0.048
*p*	**0.015**	0.092	0.109	0.152
Response inhibition				
*β*	0.118***	0.102***	−0.006	0.015
*p*	**0.000**	**0.000**	0.866	0.656
Attentional flexibility				
*β*	0.022	0.010	0.018	0.031
*p*	0.532	0.749	0.629	0.353
Working memory				
*β*	−0.009	−0.039	0.024	0.018
*p*	0.784	0.228	0.510	0.585

Values accepted after Holm–Bonferroni correction are in bold

* *p* < 0.05; ** *p* < 0.01; *** *p* < 0.001

In the longitudinal analyses in girls, only enhanced response time variability predicted affective problems after 2.5 years in girls (*β* = 0.09, *p* = 0.005). After adjustment for baseline affective problems, this predictive effect of response time variability disappeared (*β* = 0.03, *p* = 0.29). After 5 years follow-up, affective problems in girls were predicted by decreased response inhibition (*β* = 0.12, *p* < 0.001) even after adjustment for the effect of baseline affective problems (*β* = 0.10, *p* < 0.001). Response time variability was also a predictor in girls (*β* = 0.08, *p* = 0.015), but not after adjustment for baseline affective problems (*β* = 0.05, *p* = 0.09). In boys, neurocognitive performance did neither predict affective problems after 2.5 nor 5 years.

### Error rates and affective problems

Errors on the sustained attention dot patterns task, the visual attention set shifting task and the memory search letter task were not associated with affective problems, neither at baseline, nor at follow-up after 2.5 and 5 years (*p*s > 0.05).

## Discussion

The objective of the present study was to better understand the relationship between affective problems and neurocognitive functioning in adolescent boys and girls, in cross-sectional and in longitudinal perspective. In line with our first hypothesis slower baseline speed, enhanced response time variability, deficient response inhibition and poor working memory were cross-sectionally associated with affective problems at age 10–12 years. Moreover, as we expected, this was the case for girls but not for boys. With respect to our second hypothesis, enhanced response time variability indeed predicted affective problems at 2.5- and 5-year follow-up. Deficient response inhibition also predicted affective problems at 5-year follow-up, but not at 2.5-year follow-up.

The present findings suggest that enhanced response time variability is associated with affective problems in girls, but not in boys, and suggest that enhanced response time variability coincides with and predicts affective problems. Therefore, response time variability may be a risk factor in the causal pathway of affective problems. The question, which underlying mechanism explains this relation remains. Previous research in adults already showed that response time variability on sustained attention tasks tends to increase more quickly in depressed patients than in healthy participants, and several explanations have been offered. Depressed patients were suggested to have an increased susceptibility to fatigue, may be lacking in sustained motivation, may be unable to maintain concentration, and may be lacking physiological preparedness to react [50, 51]. An additional explanation may be that enhanced variability in response time is a neurocognitive marker of stress reactivity. Stress reactivity is a risk factor for depression that is mediated by genetic risk but primarily influenced by environmental factors, i.e., chronic stress in childhood [52, 53]. A recent study in adolescents with ADHD showed that response time variability was associated with increased cortisol levels after stress [54], which was consistent with earlier research in depressed patients showing cognitive deficits related to cortisol secretion [55, 56]. Additionally, response time variability has been associated with poor attentional control in the prefrontal cortex (PFC) [57, 58]. Under stress, PFC activity is closely related to activity of the hypothalamic pituitary adrenal (HPA) axis, and high levels of adrenergic activation were shown to have a detrimental effect on attention performance [54, 59, 60]. So, response time variability and increased stress reactivity, which is a neurocognitive risk marker for depression, may be closely related.

The present study shows that only in girls, enhanced response time variability is associated with affective problems. This is an interesting result since twice as many women than men are found to suffer from depression, and a gender difference starts to emerge in adolescence [29, 61, 62]. Furthermore, cortisol reactivity to stress differs between boys and girls [63] and daughters, not sons, of depressed parents showed aberrant cortisol reactivity to stress [64]. Sex hormone levels may play a role because these have been associated with the incidence of depression and may also influence stress reactivity by modulating the maturation, activation and feedback of the HPA axis [65–67]. Future research should clarify the potential role of cortisol reactivity to stress in the causal pathway that links response time variability to affective problems in adolescence.

Remarkably, response inhibition did not predict affective problems in girls after 2.5 years but strongly predicted affective problems after 5 years. This may suggest a time lag in the prediction of affective problems and may suggest that impaired response inhibition in early adolescence is a prodromal factor for affective problems in late adolescence and not for affective problems in early adolescence. Late-onset depression (late adolescence and adulthood) differs from early-onset depression (childhood and early adolescence) in etiology and phenomenology. Late-onset depression is more likely attributed to genetic factors and favorable in terms of less symptom severity, less recurrences and less invalidation [68, 69].

It has been suggested that unlike response time variability, response inhibition in depression is not associated with cortisol but it may be mediated by another neurochemical system [70]. Therefore, response inhibition may be part of another causal pathway than response time variability in the prediction of affective problems. Response inhibition has been claimed to rely on dopaminergic pathways [71–73] that emerge mainly from the interaction of the prefrontal cortex (PFC) and the basal ganglia [74–76]. These dopaminergic pathways are involved in self-control and reward sensitivity that play a role in adolescent depression. In those who show impaired dopamine-related functioning in the PFC, the complexity of social relationships in late adolescence may inflict depressive symptoms [77–79]. Response inhibition may be a neurocognitive marker of the dopamine-related increased risk for depressive symptoms. Response inhibition was only predictive in girls, not in boys. This specificity is remarkable but consistent with a recent study that showed a similar gender effect in patients with autism spectrum disorders, in which response inhibition was only impaired in female patients [80].

In terms of state or trait, the present findings suggest that response time variability and response inhibition that predicted affective problems may at least partly be a trait factor. This is in line with previous studies that showed that response time variability in children and adolescents was of modest stability [81] and that response inhibition is stable in test–retest [85]. The present study shows that baseline response speed and working memory were only coincidentally associated with affective problems in the present study. Therefore, these neurocognitive functions may be state factors and may not be risk factors in the causal pathway of affective problems. Previous studies already showed that neurocognitive impairment was at least partly a state effect [16, 17, 19, 20] or at least partly a trait effect [10–13]. A quantification on a continuum between fully state and fully trait may be more appropriate to measure the strength of state–trait characteristics. The state–trait issue is only partly covered by the present study. Follow-up data on the neurocognitive variables were not available, only for affective problems. Therefore, the (in)stability of neurocognitive factors over time is not examined.

Neurocognitive functioning was only associated with affective problems in girls, but not in boys. Thus, gender matters. Actually, in the baseline age-cohort of 10–12 years, we identified gender differences in neurocognitive functioning, whereas the severity of affective problems did not differ between boys and girls until the age of 12.5 years (i.e., wave two and three). The latter is consistent with a previous study that concluded that prevalence rates of affective problems in boys and girls start to diverge from the age of 13 [82]. So far, little attention has been paid to the role of gender in the relationship between neurocognitive impairment and depression. An empirical study confirmed the importance of gender stratification by showing that female depressed patients had more cognitive interference and a lower visual recall than male depressed patients. These gender differences were suggested to be associated with gender differences in the laterality of hippocampal activity and in prefrontal cortex functioning [83]. A recent review proposed a vulnerability–stress model that integrated affective, biological and cognitive models to explain the emergence of the gender difference in (subclinical) depression [84]. The described cognitive vulnerabilities include rumination and depressive attributional styles. However, our results suggest that more general neurocognitive vulnerability plays a role in the gender-specific developmental trajectory of depression.

The question arises whether neurocognitive functions associated with affective problems are specific to affective problems or related to psychiatric problems in general. Increased response time variability and impaired response inhibition have also been associated with ADHD [85, 86] and bipolar disorder [87]. Impaired baseline response speed had previously been found in girls with MDD [4], but seems not specific to depression [50]. It has also been shown in children with ADHD, using exactly the same neurocognitive task [88]. With respect to working memory problems, previous research on the TRAILS sample provided evidence that working memory impairment might be a potential marker of the severity of more general (externalizing) problem behavior [38]. Additionally, it was suggested that children with only internalizing problems (which contained affective problems, anxiety problems and somatic problems together) did not differ on working memory capacity from children without problem behaviors [38]. Since the latter was in contrast with our present findings, we conducted post hoc stratified analyses within the construct of internalizing problems. Those results suggest that impaired working memory is specific to affective problems (Kendall’s tau = 0.043, *p* < 0.005) and not associated with anxiety problems (Kendall’s tau = 0.001, *p* = 0.93) or somatic problems (Kendall’s tau = −0.022, *p* = 0.13).

The present findings must be viewed in light of some limitations. We based our data on affective problems on self-report rather than on psychiatric interviews. However, the content validity of the YSR scores on the affective problems is good [46]. Moreover, self-report has been shown to be the most relevant and valid measure to assess affective problems in adolescents, especially in girls [89]. Further, we did not include specific cognitive measures of processing emotional information, and cannot directly compare the contribution of processing of emotionally neutral and loaded stimuli.

In summary, our results add to the literature on depression in that aspects of information processing as reflected in response time variability and response inhibition are found to predict affective problems after 2.5 and 5 years in adolescent girls only. Further research into the causal pathway of affective problems and neurocognitive function should take account of gender effects.
